# Lymphatic and Angiogenic Candidate Genes Predict the Development of Secondary Lymphedema following Breast Cancer Surgery

**DOI:** 10.1371/journal.pone.0060164

**Published:** 2013-04-16

**Authors:** Christine Miaskowski, Marylin Dodd, Steven M. Paul, Claudia West, Deborah Hamolsky, Gary Abrams, Bruce A. Cooper, Charles Elboim, John Neuhaus, Brian L. Schmidt, Betty Smoot, Bradley E. Aouizerat

**Affiliations:** 1 Department of Physiological Nursing, University of California San Francisco, San Francisco, California, United States of America; 2 Department of Physiological Nursing, Helen Diller Family Comprehensive Cancer Center, University of California San Francisco, San Francisco, California, United States of America; 3 Department of Neurology, University of California San Francisco, San Francisco, California, United States of America; 4 Department of Community Health Systems, University of California San Francisco, San Francisco, California, United States of America; 5 Redwood Regional Medical Group, Santa Rosa, California, United States of America; 6 Department of Epidemiology and Biostatistics, University of California San Francisco, San Francisco, California, United States of America; 7 Department of Oral and Maxillofacial Surgery, New York University, New York, New York, United States of America; 8 Department of Physical Therapy and Rehabilitation Science, University of California San Francisco, San Francisco, California, United States of America; 9 Department of Physiological Nursing, Institute for Human Genetics, University of California San Francisco, San Francisco, California, United States of America; Sudbury Regional Hospital, Canada

## Abstract

The purposes of this study were to evaluate for differences in phenotypic and genotypic characteristics in women who did and did not develop lymphedema (LE) following breast cancer treatment. Breast cancer patients completed a number of self-report questionnaires. LE was evaluated using bioimpedance spectroscopy. Genotyping was done using a custom genotyping array. No differences were found between patients with (n = 155) and without LE (n = 387) for the majority of the demographic and clinical characteristics. Patients with LE had a significantly higher body mass index, more advanced disease and a higher number of lymph nodes removed. Genetic associations were identified for four genes (i.e., lymphocyte cytosolic protein 2 (rs315721), neuropilin-2 (rs849530), protein tyrosine kinase (rs158689), vascular cell adhesion molecule 1 (rs3176861)) and three haplotypes (i.e., Forkhead box protein C2 (haplotype A03), neuropilin-2 (haplotype F03), vascular endothelial growth factor-C (haplotype B03)) involved in lymphangiogensis and angiogenesis. These genetic associations suggest a role for a number of lymphatic and angiogenic genes in the development of LE following breast cancer treatment.

## Introduction

Lymphedema (LE) following treatment for breast cancer is the most common form of LE in the industrialized world [1]. LE is caused by a reduction in lymph transport capacity associated with the cancer and its treatment (e.g., surgery, radiation therapy (RT), chemotherapy (CTX)) and results in the accumulation of protein-rich fluid in the interstitial space. LE results in disfigurement, discomfort, and functional impairments. In addition, LE may precipitate cellulitis and lymphangitis [2].

The exact prevalence of LE in breast cancer survivors is unknown with estimates ranging from 3% to 87% [3]. This wide variation is due to differences in measurement methods, inconsistencies in diagnostic criteria, as well as variations in sample characteristics, timing of measurements, and duration of follow-up. However, as noted by Armer [3], if the incidence of LE is conservatively set at 20%, more than 500,000 breast cancer survivors in the United States are affected by this condition.

One of the major goals of LE research is the identification of women at greatest risk for the development of this condition following breast cancer treatment. Risk factors evaluated in the majority of studies done to date focused on patient, disease, and treatment characteristics. However, in four large scale prospective cohort studies [4]–[7], the factors associated with increased risk for LE were not concordant. In a study of 1,287 women (104 were diagnosed with LE) [5], risk factors for LE included: higher stage of disease, increased number of lymph nodes removed, removal of tumor positive nodes, receipt of adjuvant CTX, higher body mass index (BMI), and poorer health status. In a second study of 997 women (133 had LE) [4], risk factors for LE included: being African American, being better educated, higher stage of disease, and removal of at least one lymph node. In a population-based sample of 631 women (237 had LE) [6], hazard ratios for the development of LE were increased following axillary lymph node dissection (ALND) and receipt of CTX. Finally, in a sample of breast cancer patients (n = 2,431) from the Women's Healthy Eating and Living Well Study [7], women with LE (28.5% of the sample) were diagnosed at a younger age, had a higher BMI, had a larger tumor size, had more lymph nodes removed, were more likely to have a mastectomy with radiation therapy (RT) and were more likely to have CTX. While all four studies evaluated a large number of risk factors, the diagnosis of LE relied on patient self-report [5]–[7] or medical record data [4].

While the phenotypic characterization of risk for LE following breast cancer treatment warrants additional investigation, recent evidence suggests that some of the variation in the occurrence and time to onset of LE may be related to genomic determinants [8], [9]. These two studies evaluated candidate genes that are known to play a role in lymphatic development [10]–[15] or were identified in studies of primary LE which are Mendelian inherited disorders. In a study that evaluated hepatocyte growth factor (HGF) and the high affinity hepatocyte growth factor receptor (MET) in 59 women with breast cancer related LE [9], mutations leading to truncation or missense changes in evolutionarily conserved residues of HGF and MET were identified. In a case control study of 188 women (80 diagnosed with LE) [8], mutations in GJC2 that encodes for connexin 47 were identified in women with LE. Taken together, these findings suggest that additional studies are warranted to determine the phenotypic and genotypic factors associated with increased risk for LE following breast cancer treatment. Therefore, the purposes of this study were to evaluate for differences in phenotypic and genotypic characteristics in women who did and did not develop LE following breast cancer treatment.

## Methods

### Study Samples and Procedures

Demographic, clinical, and genomic data from a cross-sectional study (i.e., LE Study (NR0101282)) and a longitudinal study (i.e., Breast Symptoms Study (CA107091 and CA118658)) were combined for these analyses. Both studies used the same subjective and objective measures. Both studies were approved by the UCSF Committee on Human Research and the CTSI Clinical Research Center Advisory Committee.

#### LE Study

The LE study used a cross-sectional design to evaluate for differences in phenotypic and genotypic characteristics in women with (n = 74) and without LE (n = 71). Women who were ≥18 years of age, ≥6 months post-treatment for unilateral breast cancer, and with or without upper extremity LE were recruited. Women were excluded for bilateral breast cancer, current upper extremity infection, lymphangitis, preexisting LE, current breast cancer, or contraindications to bioimpedance spectroscopy (BIS) testing. Women were recruited through the National Lymphedema Network website, San Francisco Bay area hospitals, and breast cancer or LE support groups and conferences. Women were evaluated in the Clinical Research Center at UCSF. After obtaining written informed consent, women completed the study questionnaires. Following the completion of the questionnaires, the research staff performed the objective measurements: height, weight, and BIS. A blood sample was drawn for genomic analyses.

#### Breast Symptoms Study

The Breast Symptoms Study used a prospective, longitudinal design to evaluate neuropathic pain and LE following breast cancer surgery. Women were recruited from Breast Care Centers located in a Comprehensive Cancer Center, two public hospitals, and four community practices. Patients were eligible to participate if they were: adult women (≥18 years) who would undergo breast cancer surgery on one breast; able to read, write, and understand English; agreed to participate, and gave written informed consent. Patients were excluded if they were: having breast cancer surgery on both breasts and/or had distant metastasis at the time of diagnosis. A total of 516 patients were approached to participate, 410 were enrolled in the study (response rate 79.4%), and 398 completed the preoperative assessment. The major reasons for refusal were: too busy, overwhelmed with the cancer diagnosis, or insufficient time available to do the baseline assessment prior to surgery. During the patient's preoperative visit, a clinician explained the study, determined the patient's willingness to participate, and introduced the patient to the research nurse. The research nurse met with the woman, determined eligibility, and obtained written informed consent prior to surgery.

After obtaining written informed consent, the patient completed the enrollment questionnaires prior to surgery. Following the completion of the questionnaires, the research nurse performed the objective measurements: height, weight, and BIS. A blood sample was drawn for genomic analyses. Patients were contacted two weeks after surgery to schedule the first post-surgical appointment. The research nurse met with the patients either in their home or in the Clinical Research Center at 1, 2, 3, 4, 5, 6, 8, 10, and 12 months after surgery. In the second through fifth years of the study, patients were seen every four months. During each of the study visits, the women completed the study questionnaires and had the objective measures done by the research nurse.

### Subjective Measures

A demographic questionnaire was used to obtain information on age, marital status, education, ethnicity, employment status, living situation, and financial status. Functional status was evaluated using the Karnofsky Performance Status (KPS) scale that has well established validity and reliability [16], [17]. Patients rated their functional status using the KPS scale that ranged from 30 (I feel severely disabled and need to be hospitalized) to 100 (I feel normal; I have no complaints or symptoms). Patients were asked to indicate if they exercised on a regular basis (yes/no). Clinical information was obtained from patient interviews and medical record reviews.

Self-Administered Comorbidity Questionnaire (SCQ) is a short and easily understood instrument that was developed to measure comorbidity in clinical and health service research settings [18]. The questionnaire consists of 13 common medical conditions that were simplified into language that could be understood without any prior medical knowledge. Patients were asked to indicate if they had the condition using a “yes/no” format. If they indicated that they had a condition, they were asked if they received treatment for it (yes/no; proxy for disease severity) and did it limit their activities (yes/no; indication of functional limitations). Patients were given the option to add two additional conditions not listed on the instrument. For each condition, a patient can receive a maximum of 3 points. Because there are 13 defined medical conditions and 2 optional conditions, the maximum score totals 45 points if the open-ended items are used and 39 points if only the closed-ended items are used. The SCQ has well-established validity and reliability and has been used in studies of patients with a variety of chronic conditions [18]–[22].

### Objective Measures

#### Bioimpedance Spectroscopy (BIS) of LE

BIS measurements, of the affected and unaffected arm, were done using the procedures described by Cornish and colleagues [23]–[25]. Patients were instructed not to exercise or take a sauna within 8 hours of the assessment. In addition, they were asked to refrain from drinking alcohol for 12 hours prior to the assessment. BIS measurements were taken using a single channel BIS device (i.e., SFB7 device; ImpediMed, San Diego, CA in the LE study or the Quantum X Bioelectrical Impedance Device; RJL Systems, Clinton Township, MI in the Breast Symptoms Study). Women removed all jewelry and their skin was prepped with an alcohol wipe prior to surface electrode placement. Patients lay supine on a massage table with their arms 30 degrees from the body and legs not touching for at least 10 minutes prior to the BIS measurements. Electrodes were placed on the dorsum of the wrists adjacent to the ulnar styloid process, the dorsum of the hands just proximal to the third metacarpophalangeal joint, anterior to the ankle joints between the malleoli, and over the dorsum of the feet over the third metatarsal bone just proximal to the third metatarsophalangeal joint. Two ‘measurement’ electrodes were placed at either end of the 40 cm length over which the circumference measurements were made and the ‘drive’ electrodes were placed 8 to 10 cm distal to these measurement electrodes. These electrode sites were chosen, for the segmental measurement of the arm, in preference to the standard shoulder to wrist sites so that direct comparisons could be made between the volumes measured by the circumference method and by the BIS method. Two readings of resistance were obtained from the affected and unaffected arms and averaged for subsequent analyses.

While cases and non-cases of LE were known in the LE study, for the Breast Symptoms Study, LE cases were determined based on the procedures of Cornish and colleagues [23]–[25], using all of the data obtained from each woman during her participation in the study. A woman was defined as a LE case if the resistance ratio for the untreated arm/treated arm prior to surgery was >1.139 or >1.066 for those women who had surgery on the dominant or nondominant side, respectively at any of the BIS assessments.

### Methods of Analysis for Phenotypic Data

Data were analyzed using SPSS Version 19 [26]. Descriptive statistics and frequency distributions were generated on the sample characteristics. Independent sample t-tests, Chi-square analyses, and Mann Whitney U tests were done to evaluate for differences in demographic, clinical, and genotypic characteristics between patients with and without LE. Logistic regression analyses were performed to evaluate the association between phenotypic characteristics and LE group membership.

### Methods of Analysis for Genomic Data

#### Gene Selection

Candidate genes for secondary LE include genes that cause monogenic (i.e., primary) forms of LE or genes that cause primary LE in animal models [27]–[31]. While the genetic causes of primary LE might be due to ablative mutations or variations in these candidate genes, secondary LE may be caused by modest functional variations that do not result in critical loss of gene function. Candidate genes for primary LE include angiopoeitin-2 (ANGPT2), elastin microfibril interfacer (EMILIN1), Forkhead box protein C2 (FOXC2), hepatocye growth factor (HGF), lymphocyte cytosolic protein 2 (LCP2), lymphatic vessel endothelial hyaluronan receptor 1 (LYVE1, XLKD1), hepatocyte growth factor receptor (MET), neuropilin-2 (NRP2), Prospero-related homeobox 1 (PROX1), ROR orphan receptor C (RORC), SpSRY-box 17 (SOX17), protein tyrosine kinase (SYK), vascular cell adhesion molecule 1 (VCAM1), and vascular endothelial growth factor-B (VEGFB), -C (VEGFC), -D (VEGFD), -receptor 2 (VEGFR2), and -receptor 3 (VEGFR3).

#### Blood collection and genotyping

Genomic DNA was extracted from archived buffy coats using the PUREGene DNA Isolation System (Invitrogen, Carlsbad, CA). Of the 543 patients recruited for this study, DNA was recovered from the archive buffy coat of 407 patients (i.e., 110 with and 297 without LE) who provided a blood sample. No differences were found in any demographic and clinical characteristics between patients who did and did not choose to participate in the studies or between those patients who did and did not provide a blood sample for genomic analyses.

Genotyping was performed blinded to LE status and positive and negative controls were included. DNA was quantitated with a Nanodrop Spectrophotometer (ND-1000) and normalized to a concentration of 50 ng/μL (diluted in 10 mM Tris/1 mM EDTA). Samples were genotyped using the GoldenGate genotyping platform (Illumina, San Diego, CA) and processed according to the standard protocol using GenomeStudio (Illumina, San Diego, CA). Signal intensity profiles and resulting genotype calls for each single nucleotide polymorphism (SNP) were visually inspected by two blinded reviewers. Disagreements were adjudicated by a third reviewer.

#### SNP Selection

A combination of tagging SNPs and literature driven SNPs (i.e., SNPs reported as being associated with LE) were selected for analysis. Tagging SNPs were required to be common (i.e., estimated to have a minor allele frequency ≥.05) in public databases (e.g., HapMap). In order to ensure robust genetic association analyses, quality control filtering of SNPs was performed. SNPs with call rates of <95% or Hardy-Weinberg p-values of <.001 were excluded.

As shown in Table 1, a total of 157 SNPs among the 17 candidate genes (ANGPT2: 25 SNPs, FOXC2: 3 SNPs; HGF: 3 SNPs; LCP2: 11 SNPs; LYVE1: 1 SNP; MET: 15 SNPs; NRP2: 32 SNPs; PROX1: 3 SNPs; RORC: 4 SNPs; SOX17:1 SNP; SYK: 19 SNPs; VCAM1: 7 SNPs; VEGFB: 1 SNP; VEGFC: 8 SNPs; VEGFD: 4 SNPs; VEGFR2: 12 SNPs, VEGFR3: 8 SNPs) passed all quality control filters and were included in the genetic association analyses. The one SNP used to evaluate genetic variation in EMILIN1 did not pass the quality control filters. Potential functional roles of SNPs associated with LE were examined using PUPASuite 2.0 [32], a comprehensive search engine that predicts a series of functional effects (i.e., non-synonymous changes, altered transcription factor binding sites, exonic splicing enhancing or silencing, splice site alterations, microRNA target alterations).

**Table 1:** Lymphatic and angiogenic genes and single nucleotide polymorphisms analyzed for lymphedema versus no lymphedema.

Gene	SNP	Position	Chr	MAF	Alleles	Chi Square	p-value	Model
ANGPT2	rs2916716	6344643	8	.287	T>A	.380	.827	A
ANGPT2	rs2442468	6351358	8	.493	G>C	.073	.964	A
ANGPT2	rs2515409	6352330	8	.146	T>C	.131	.937	A
ANGPT2	rs2515413	6353028	8	.205	T>C	1.464	.481	A
ANGPT2	rs2442636	6354369	8	.446	T>C	.346	.841	A
ANGPT2	rs2442631	6356184	8	.428	G>A	.096	.953	A
ANGPT2	rs1982386	6358884	8	.314	G>A	.542	.762	A
ANGPT2	rs2515462	6371007	8	.287	G>A	2.660	.264	A
ANGPT2	rs6990020	6373612	8	.380	T>C	FE	.040	D
ANGPT2	rs2515466	6373694	8	.231	G>A	.230	.892	A
ANGPT2	rs2442608	6374028	8	.447	A>G	.985	.611	A
ANGPT2	rs734701	6375655	8	.329	T>C	1.570	.456	A
ANGPT2	rs2515477	6376048	8	.131	C>T	.904	.636	A
ANGPT2	rs12674822	6376624	8	.440	G>T	.865	.649	A
ANGPT2	rs2515483	6379677	8	.353	G>C	.644	.725	A
ANGPT2	rs17552444	6381587	8	.269	A>G	.731	.694	A
ANGPT2	rs11989215	6383317	8	.338	A>G	.371	.831	A
ANGPT2	rs11989242	6383428	8	.478	G>A	.567	.753	A
ANGPT2	rs11137037	6383590	8	.326	A>C	3.009	.222	A
ANGPT2	rs17623313	6383749	8	.431	C>T	.150	.928	A
ANGPT2	rs13269021	6384171	8	.270	G>T	.176	.916	A
ANGPT2	rs1823375	6384406	8	.349	C>G	1.537	.464	A
ANGPT2	rs13255574	6386085	8	.204	C>T	.208	.901	A
ANGPT2	rs2922869	6387201	8	.349	A>G	1.543	.462	A
ANGPT2	rs2515488	6390414	8	.458	A>C	2.811	.245	A
	HapA04					.119	.942	
	HapA06					.533	.766	
	HapB01					.093	.955	
	HapB04					.203	.903	
	HapC01					3.938	.140	
	HapC04					2.438	.295	
	HapD01					1.570	.456	
	HapD02					1.087	.581	
	HapD03					.797	.671	
	HapE01					1.816	.403	
	HapE02					.682	.711	
	HapE03					.860	.650	
	HapF01					3.375	.185	
	HapF07					.158	.924	
	HapG01					.677	.713	
	HapG02					1.392	.499	
	HapG03					.295	.863	
	HapH02					1.523	.467	
	HapH03					.202	.904	
FOXC2	rs34221221	85157931	16	.452	T>C	2.830	.243	A
FOXC2	rs11640590	85159945	16	*0.000*	C>A	All patients were homozygous for CC		
FOXC2	rs1035550	85160208	16	.087	C>T	3.934	.140	A
	HapA01					2.830	.243	
	HapA03					6.214	.045	
HGF	rs5745692	81196202	7	.*033*	G>C	n/a	n/a	n/a
HGF	rs757830	81200320	7	.221	T>C	1.998	.368	A
HGF	rs5745616	81236292	7	.222	G>A	1.315	.518	A
LCP2	rs3789184	169611615	5	.494	T>C	2.794	.247	A
LCP2	rs572192	169621175	5	.410	A>G	FE	.047	D
LCP2	rs10475933	169622825	5	.285	C>T	.409	.815	A
LCP2	rs2338872	169625132	5	.366	A>G	1.492	.474	A
LCP2	rs315745	169630285	5	.459	T>C	.431	.806	A
LCP2	rs2271146	169634968	5	.466	G>T	.119	.942	A
LCP2	rs2338873	169641269	5	.141	G>A	3.164	.206	A
LCP2	rs315721	169647616	5	.305	A>G	FE	.005	D
LCP2	rs182618	169655691	5	.278	A>G	4.040	.133	A
LCP2	rs6866733	169655807	5	.197	C>T	FE	.026	D
LCP2	rs315730	169656902	5	.434	A>T	.644	.725	A
	HapA03					.510	.775	
	HapA04					4.304	.116	
LYVE1	rs17318858	10536263	11	.176	T>C	.547	.761	A
MET	rs714180	116106238	7	.488	G>A	.981	.612	A
MET	rs38841	116107162	7	.362	A>G	.001	1.000	A
MET	rs39747	116108275	7	.441	T>C	2.782	.249	A
MET	rs38845	116109038	7	.456	G>A	4.550	.103	A
MET	rs39748	116114666	7	.442	G>C	1.177	.555	A
MET	rs38849	116119775	7	.249	G>C	2.414	.299	A
MET	rs2237710	116124588	7	.289	T>G	1.666	.435	A
MET	rs38850	116124885	7	.246	G>A	2.491	.288	A
MET	rs11762213	116126518	7	.053	G>A	1.434	.488	A
MET	rs2299437	116128724	7	.244	G>A	1.244	.537	A
MET	rs38857	116152649	7	.275	C>T	1.954	.376	A
MET	rs2402118	116215809	7	.338	C>A	1.915	.384	A
MET	rs193686	116218663	7	.287	T>C	1.569	.456	A
MET	rs2023748	116223258	7	.447	G>A	2.968	.227	A
MET	rs1621	116224842	7	.318	A>G	1.909	.385	A
	HapA01					2.427	.297	
	HapA03					4.016	.134	
	HapB02					2.545	.280	
	HapC02					3.296	.192	
	HapC06					1.704	.427	
	HapC07					2.003	.367	
NRP2	rs1517527	206252547	2	.408	C>T	1.126	.569	A
NRP2	rs6711044	206252910	2	.479	T>C	.413	.813	A
NRP2	rs1400733	206253168	2	.432	C>G	.329	.848	A
NRP2	rs3806577	206254395	2	.412	A>G	.291	.865	A
NRP2	rs861078	206260223	2	.351	A>G	1.320	.517	A
NRP2	rs849530	206264049	2	.482	T>G	FE	.042	R
NRP2	rs950219	206268858	2	.308	G>A	3.601	.165	A
NRP2	rs3771052	206269672	2	.293	G>A	2.566	.272	A
NRP2	rs849556	206271503	2	.305	G>A	1.858	.395	A
NRP2	rs863707	206284428	2	.445	T>G	.678	.712	A
NRP2	rs849525	206301486	2	.466	G>A	.635	.728	A
NRP2	rs3771033	206304055	2	.326	G>A	3.654	.161	A
NRP2	rs849523	206304181	2	.279	C>T	1.299	.522	A
NRP2	rs1983343	206304908	2	.343	A>G	1.258	.533	A
NRP2	rs849584	206309961	2	.318	G>T	2.925	.232	A
NRP2	rs3771021	206318233	2	.405	C>T	.723	.697	A
NRP2	rs849563	206318747	2	.187	A>C	FE	.012	R
NRP2	rs1996412	206320830	2	.473	A>G	4.227	.121	A
NRP2	rs2241156	206323209	2	.358	G>C	1.564	.457	A
NRP2	rs2241155	206323311	2	.358	C>T	.293	.864	A
NRP2	rs3771016	206323784	2	.418	G>A	.005	.998	A
NRP2	rs3771010	206331839	2	.477	G>C	.219	.896	A
NRP2	rs867344	206335660	2	.346	C>T	.783	.676	A
NRP2	rs3771004	206339132	2	.305	G>A	.006	.997	A
NRP2	rs16837637	206339499	2	.402	G>A	.417	.812	A
NRP2	rs16837641	206343114	2	.371	G>A	FE	.009	R
NRP2	rs2241153	206344368	2	.383	G>T	1.748	.417	A
NRP2	rs2160328	206350582	2	.374	C>T	2.150	.341	A
NRP2	rs4675542	206353492	2	.213	G>C	1.940	.379	A
NRP2	rs10932125	206360545	2	.456	G>C	3.922	.141	A
NRP2	rs3755232	206361208	2	.253	A>G	3.096	.213	A
NRP2	rs15994	206370542	2	.377	C>G	1.106	.575	A
	HapA01					5.793	.055	
	HapA06					2.709	.258	
	HapB01					3.427	.180	
	HapB04					.631	.730	
	HapB05					1.340	.512	
	HapC01					.163	.922	
	HapC02					.367	.832	
	HapC03					3.431	.180	
	HapD01					.137	.934	
	HapD06					.430	.807	
	HapE01					3.083	.214	
	HapE02					.372	.830	
	HapE03					.006	.997	
	HapF01					8.937	.011	
	HapF03					1.574	.455	
	HapF06					1.426	.490	
PROX1	rs340874	212225879	1	.499	A>G	1.919	.383	A
PROX1	rs340839	212228443	1	.483	C>T	2.961	.227	A
PROX1	rs726334	212246741	1	.284	C>T	.726	.696	A
RORC	rs9826	150045523	1	.371	A>G	.097	.953	A
RORC	rs939595	150050312	1	.381	C>A	.232	.891	A
RORC	rs7540530	150057482	1	.466	G>A	.420	.811	A
RORC	rs11204894	150059798	1	.225	G>T	3.952	.139	A
	HapA01					.420	.811	
	HapA03					.624	.732	
	HapA06					4.005	.135	
SOX17	rs12541742	55533707	10	.210	C>T	FE	.008	D
SYK	rs1319677	92605820	9	.484	T>C	.171	.918	A
SYK	rs1333633	92607393	9	.416	T>C	.835	.659	A
SYK	rs3789889	92611162	9	.339	A>G	2.008	.366	A
SYK	rs290237	92614471	9	.204	A>G	.034	.983	A
SYK	rs4744505	92619818	9	.391	G>A	.140	.932	A
SYK	rs2065583	92632383	9	.151	G>C	1.174	.556	A
SYK	rs290213	92635628	9	.143	A>G	1.116	.572	A
SYK	rs1870660	92637826	9	.163	C>G	.682	.711	A
SYK	rs1864202	92641776	9	.261	C>A	2.573	.276	A
SYK	rs17489214	92642959	9	.186	G>A	.342	.843	A
SYK	rs2035073	92649240	9	.328	T>C	.093	.955	A
SYK	rs11787537	92656440	9	.185	G>A	.576	.750	A
SYK	rs10993726	92660569	9	.214	C>T	.678	.713	A
SYK	rs9695993	92663585	9	.123	A>C	.742	.690	A
SYK	rs290229	92674234	9	.259	C>T	3.746	.154	A
SYK	rs10761395	92682718	9	.300	T>C	2.009	.366	A
SYK	rs290254	92691706	9	.408	G>C	.078	.962	A
SYK	rs158689	92697582	9	.456	A>T	FE	.039	R
SYK	rs1049164	92698027	9	.178	G>A	2.849	.241	A
	HapA01					.204	.903	
	HapA04					.688	.709	
	HapB01					.617	.735	
	HapB02					.161	.923	
	HapB03					.029	.985	
	HapC01					1.608	.448	
	HapD01					9.769	.008	
	HapD03					.093	.955	
	HapE01					3.746	.154	
	HapE02					.024	.988	
	HapE03					2.009	.366	
	HapF01					5.000	.082	
	HapF03					5.254	.072	
VCAM1	rs1409419	100955984	1	.498	T>C	1.095	.578	A
VCAM1	rs3176860	100959807	1	.412	A>G	.880	.644	A
VCAM1	rs3176861	100959909	1	.201	C>T	FE	.036	D
VCAM1	rs3917012	100968247	1	.350	T>G	3.381	.184	A
VCAM1	rs3181088	100971296	1	.167	C>T	1.520	.468	A
VCAM1	rs3176877	100975983	1	.396	T>A	2.366	.306	A
VCAM1	rs3176879	100976415	1	.061	A>G	1.579	.454	A
	HapA01					1.579	.454	
	HapA05					.825	.662	
	HapB01					8.241	.016	
	HapB02					2.366	.306	
VEGFB	rs3741403	63756105	11	.433	G>A	.553	.759	A
VEGFC	rs1485762	177844725	4	.310	C>T	5.000	.082	A
VEGFC	rs7664413	177845701	4	.206	C>T	6.364	.041	A
VEGFC	rs6828869	177847127	4	.451	C>G	.329	.848	A
VEGFC	rs1485766	177847878	4	.469	A>C	.219	.896	A
VEGFC	rs3775202	177848205	4	.494	A>G	2.226	.329	A
VEGFC	rs3775195	177858104	4	.238	C>A	1.112	.573	A
VEGFC	rs3775194	177860871	4	.407	C>G	.482	.786	A
VEGFC	rs1485765	177864946	4	.156	A>G	1.453	.484	A
	HapA01					.297	.862	
	HapA05					6.173	.046	
	HapB01					2.463	.292	
	HapB03					7.194	.027	
	HapB04					1.254	.534	
VEGFD	rs6527518	15276100	X	.435	G>T	.009	.996	A
VEGFD	rs6418686	15297927	X	.329	T>C	.116	.944	A
VEGFD	rs4830939	15309204	X	.324	G>A	.182	.913	A
VEGFD	rs6632528	15312319	X	.258	T>C	.264	.876	A
	HapA01					.001	.999	
	HapA02					.474	.789	
	HapA03					.325	.850	
VEGFR2	rs12642307	55646938	4	.256	T>C	2.524	.283	A
VEGFR2	rs1531289	55649989	4	.288	G>A	.111	.946	A
VEGFR2	rs7671745	55651593	4	.329	G>A	.557	.757	A
VEGFR2	rs6828477	55661558	4	.398	T>C	.801	.670	A
VEGFR2	rs2168945	55662240	4	.327	T>G	1.008	.604	A
VEGFR2	rs1870377	55667731	4	.254	T>A	3.543	.170	A
VEGFR2	rs2034965	55672557	4	.278	G>A	.923	.630	A
VEGFR2	rs11941492	55672967	4	.243	C>T	2.307	.316	A
VEGFR2	rs10020464	55673827	4	.330	C>T	FE	.025	D
VEGFR2	rs11133360	55677509	4	.455	T>C	FE	.032	R
VEGFR2	rs1531290	55681319	4	.437	A>G	.836	.658	A
VEGFR2	rs12502008	55685799	4	.397	G>T	1.751	.417	A
	HapA01					.386	.825	
	HapA02					.604	.739	
	HapA03					.111	.946	
	HapB02					3.331	.189	
	HapB03					1.096	.578	
	HapB04					.716	.699	
	HapC01					4.155	.125	
	HapC03					.278	.870	
	HapC04					7.730	.021	
VEGFR3	rs2242216	179974097	5	.430	G>A	.137	.934	A
VEGFR3	rs400330	179974268	5	.375	T>C	.274	.872	A
VEGFR3	rs307823	179984714	5	.271	A>G	1.617	.446	A
VEGFR3	rs3797102	179987794	5	.392	T>C	.255	.880	A
VEGFR3	rs2290983	179991569	5	.453	T>C	.708	.702	A
VEGFR3	rs10085109	179993410	5	.*484*	C>G	n/a	n/a	n/a
VEGFR3	rs11748431	180001347	5	.237	G>A	2.704	.259	A
VEGFR3	rs307814	180006854	5	.387	C>T	.179	.914	A
	HapA01					.276	.871	
	HapA03					.137	.934	
	HapB02					FE	.643	
	HapB03					3.693	.158	
	HapB04					.453	.797	
	HapC01					.556	.757	
	HapC02					3.163	.206	
	HapC03					.490	.783	

Abbreviations: A =  Additive model; ANGPT2  =  angiopoeitin-2; Chr  =  chromosome; D =  Dominant model; FOXC2 – forkhead box C2; HGF  =  hepatocyte growth factor; LCP2  =  lymphocyte cytosolic protein 2; LYVE1  =  lymphatic vessel endothelial hyaluronan receptor 1 (XLKD1); MAF  =  minor allele frequency; MET  =  hepatocyte growth factor receptor; n/a  =  not assayed because SNP violated Hardy-Weinberg expectations (p<.001); NRP2 – neuropilin-2; PROX1  =  prospero-related homeobox 1; R =  Recessive model; RORC  =  ROR orphan receptor C; SNP  =  single nucleotide polymorphism; SOX17  =  SpSRY-box 17; SYK  =  protein tyrosine kinase; VCAM1  =  vascular cell adhesion molecule 1; VEGFB  =  vascular endothelial growth factor B; VEGFC  =  vascular endothelial growth factor C; VEGFD  =  vascular endothelial growth factor D; VEGFR2  =  vascular endothelial growth factor receptor 2; VEGFR3  =  vascular endothelial growth factor receptor 3.

#### Statistical Analyses

Allele and genotype frequencies were determined by gene counting. Hardy-Weinberg equilibrium was assessed by the Chi-square or Fisher Exact tests. Measures of linkage disequilibrium ((LD) i.e., D' and r^2^) were computed from the participants' genotypes with Haploview 4.2. LD-based haplotype block definition was based on D' confidence interval [33].

For SNPs that were members of the same haploblock, haplotype analyses were conducted in order to localize the association signal within each gene and to determine if haplotypes improved the strength of the association with the phenotype. Haplotypes were constructed using the program PHASE version 2.1 [34]. In order to improve the stability of haplotype inference, the haplotype construction procedure was repeated five times using different seed numbers with each cycle. Only haplotypes that were inferred with probability estimates of ≥.85, across the five iterations, were retained for downstream analyses. Haplotypes were evaluated assuming a dosage model (i.e., analogous to the additive model).

Ancestry informative markers (AIMS) were used to minimize confounding due to population stratification [35]–[37]. Homogeneity in ancestry among participants was verified by principal component analysis [38], using Helix Tree (Golden Helix, Bozeman, MT). Briefly, the number of principal components (PCs) was sought which distinguished the major racial/ethnic groups in the sample by visual inspection of scatter plots of orthogonal PCs (i.e., PC 1 versus PC2, PC2 versus PC3). This procedure was repeated until no discernible clustering of patients by their self-reported race/ethnicity was possible (data not shown). One hundred and six AIMs were included in the analysis. The first three PCs were selected to adjust for potential confounding due to population substructure (i.e., race/ethnicity) by including the three covariates in all regression models.

For association tests, three genetic models were assessed for each SNP: additive, dominant, and recessive. Barring trivial improvements (i.e., delta <10%), the genetic model that best fit the data, by maximizing the significance of the p-value, was selected for each SNP. Logistic regression analysis, that controlled for significant covariates, as well as genomic estimates of and self-reported race/ethnicity, was used to evaluate the association between genotype and LE group membership. A backwards stepwise approach was used to create the most parsimonious model. Genetic model fit and both unadjusted and covariate-adjusted odds ratios were estimated using STATA version 9 [39].

As was done in our previous studies [40]–[42], based on recommendations in the literature [43], [44], the implementation of rigorous quality controls for genomic data, the non-independence of SNPs/haplotypes in LD, and the exploratory nature of the analyses, adjustments were not made for multiple testing. In addition, significant SNPs identified in the bivariate analyses were evaluated further using regression analyses that controlled for differences in phenotypic characteristics, potential confounding due to population stratification, and variation in other SNPs/haplotypes within the same gene. Only those SNPs that remained significant were included in the final presentation of the results. Therefore, the significant independent associations reported are unlikely to be due solely to chance. Unadjusted (bivariate) associations are reported for all SNPs passing quality control criteria in Table 1 to allow for subsequent comparisons and meta-analyses.

## Results

### Differences in demographic and clinical characteristics

As shown in Table 2, no differences were found between patients with and without LE for the majority of the demographic and clinical characteristics. Patients with LE had a significantly higher BMI and a lower KPS score, and were more likely to report lung disease. In addition, patients with LE had a higher number of lymph nodes removed, a higher number of positive nodes, more advanced disease at the time of diagnosis, were less likely to have had a SLNB, and were more likely to have had an ALND, had received CTX prior to or following surgery, and had received RT following surgery.

**Table 2:** Differences in demographic and clinical characteristics between patients with (n = 155) and without (n = 387) lymphedema.

Characteristic	No Lymphedema	Lymphedema	Statistics
	Mean (SD)	Mean (SD)	
Age (years)	54.9 (11.1)	56.2 (10.8)	NS
Education (years)	16.0 (2.7)	15.8 (2.8)	NS
Age at menopause (years)	47.8 (7.2)	46.7 (9.1)	NS
Body mass index (kg/m^2^)	26.1 (5.6)	28.2 (6.7)	p = .001
Karnofsky Performance Status score	93.3 (9.7)	91.1 (11.1)	p = .028
Comorbidity score	4.0 (2.9)	4.5 (3.3)	NS
Number of nodes removed	5.8 (6.3)	10.9 (9.0)	p<.0001
Number of positive nodes	0.7 (1.7)	1.7 (3.4)	p = .009

	% (n)	% (n)	
Ethnicity			
White	68.8 (265)	72.9 (113)	
Black	7.5 (29)	9.7 (15)	NS
Asian/Pacific Islander	13.0 (50)	7.1 (11)	
Hispanic/Mixed ethnic background/Other	10.6 (41)	10.3 (16)	
Lives alone			
Yes	23.0 (88)	28.9 (44)	NS
No	77.0 (295)	71.1 (108)	
Married/partnered			
Yes	47.4 (182)	52.0 (79)	NS
No	52.6 (202)	48.0 (73)	
Employed			
Yes	51.4 (197)	49.7 (76)	NS
No	48.6 (186)	50.3 (77)	
Handedness			
Right	88.8 (341)	88.9 (136)	
Left	8.1 (31)	9.2 (14)	NS
Both	3.1 (12)	2.0 (3)	
Occurrence of comorbid conditions (% and number of women who reported each comorbid condition from the Self-Administered Comorbidity Questionnaire)			
Heart disease	5.6 (21)	6.0 (9)	NS
High blood pressure	27.0 (103)	34.9 (53)	NS
Lung disease	3.7 (14)	8.1 (12)	p = .04
Diabetes	6.6 (25)	7.4 (11)	NS
Ulcer	3.7 (14)	4.7 (7)	NS
Kidney disease	1.6 (6)	2.0 (3)	NS
Liver disease	2.1 (8)	4.8 (7)	NS
Anemia	7.2 (27)	9.5 (14)	NS
Depression	21.8 (81)	26.7 (39)	NS
Osteoarthritis	19.2 (72)	26.7 (40)	NS
Back pain	29.3 (110)	31.5 (47)	NS
Rheumatoid arthritis	3.5 (13)	4.7 (7)	NS
Diagnosed with mastitis			
Yes	13.1 (50)	11.3 (17)	NS
No	86.9 (332)	88.7 (134)	
Diagnosed with cystic breast disease			
Yes	21.5 (81)	23.3 (34)	NS
No	78.5 (295)	76.7 (112)	
Breastfed			
Yes	49.4 (190)	45.1 (69)	NS
No	50.6 (195)	54.9 (84)	
Surgery on affected breast not related to cancer			
Yes	9.3 (36)	14.8 (23)	NS
No	90.7 (351)	85.2 (132)	
Surgery to the affected arm not related to cancer			
Yes	3.1 (12)	5.2 (8)	NS
No	96.9 (375)	94.8 (147)	
Surgery on the affected hand not related to cancer			
Yes	5.2 (20)	7.1 (11)	NS
No	94.8 (367)	92.9 (144)	
Injury to the affected arm			
Yes	17.3 (67)	22.6 (35)	NS
No	82.7 (320)	77.4 (120)	
Injury to the affected hand			
Yes	17.1 (66)	17.4 (27)	NS
No	82.9 (321)	82.6 (128)	
Side of cancer surgery			
Dominant	49.9 (193)	41.9 (65)	NS
Nondominant	50.1 (194)	58.1 (90)	
Type of surgery			
Breast conservation	75.2 (291)	70.3 (109)	NS
Mastectomy	24.8 (96)	29.7 (46)	
Stage of disease			
Stage 0	18.1 (70)	5.2 (8)	
Stage I	40.1 (155)	32.9 (51)	p<.0001
Stage IIA and IIB	35.4 (137)	48.4 (75)	
Stage IIIA, IIIB, IIIC, and IV	6.5 (25)	13.5 (21)	
Sentinel lymph node biopsy			
Yes	80.9 (313)	69.7 (108)	p = .006
No	19.1 (74)	30.3 (47)	
Axillary lymph node dissection			
Yes	39.3 (152)	69.3 (106)	p<.0001
No	60.7 (235)	30.7 (47)	
Reconstruction at the time of surgery			
Yes	21.6 (68)	22.2 (18)	NS
No	78.4 (247)	77.8 (63)	
Adjuvant chemotherapy			
Yes	36.7 (142)	59.7 (92)	p<.0001
No	63.3 (245)	40.3 (62)	
Adjuvant radiation therapy			
Yes	57.1 (221)	71.0 (110)	p<.0001
No	42.9 (166)	29.0 (45)	
Combinations of treatments			
Only surgery	23.8 (92)	8.4 (13)	
Surgery and radiation therapy	39.5 (153)	32.3 (50)	p<.0001
Surgery and chemotherapy	19.1 (74)	20.6 (32)	
Surgery, radiation therapy, and chemotherapy	17.6 (68)	38.7 (60)	
Exercise on a regular basis			
Yes	73.7 (283)	75.2 (115)	NS
No	26.3 (101)	24.8 (38)	

Abbreviations: kg  =  kilograms, m^2^ – meter squared, NS  =  not significant, SD  =  standard deviation.

### Regression analyses for phenotypic predictors of LE

As shown in Table 3, two regression analyses were done to evaluate the associations between phenotypic characteristics and LE group membership. In the first regression analysis, that included BMI, stage of disease, SLNB status, number of lymph nodes removed, receipt of CTX, and receipt of RT predicted 13.8% of the variance in LE group membership (p<0.0001). The odds of developing LE increased significantly for women who had a higher BMI, had a higher stage of disease at diagnosis, had a higher number of lymph nodes removed, had received CTX prior to or following surgery, and had received RT. The odds of having LE decrease significantly in women who had a SLNB.

**Table 3:** Multiple logistic regression analyses for phenotypic predictors of the development of lymphedema.

Regression analysis WITHOUT the inclusion of genomic and self-reported race/ethnicity					
Predictor	Odds Ratio	Standard Error	95% CI	Z	p-value
BMI	1.06	0.018	1.025, 1.097	3.39	0.001
Stage of disease Stage 0 versus I Stage 0 versus II Stage 0 versus III and IV	3.23 3.18 3.62	1.590 1.622 2.151	1.234, 8.479 1.171, 8.640 1.129, 11.603	2.39 2.27 2.16	0.017 0.023 0.030
SLNB	0.42	0.116	0.243, 0.719	−3.16	0.002
Number of nodes removed	1.06	0.017	1.028, 1.093	3.70	<.0001
Any chemotherapy	1.74	0.416	1.086, 2.779	2.30	0.021
Any radiation therapy	1.94	0.452	1.224, 3.060	2.83	0.005
Overall model fit: χ^2^ = 85.32, p<0.0001, R^2^ = 0.1380					

Regression analysis WITH the inclusion of genomic and self-reported race/ethnicity					
BMI	1.05	0.022	1.012, 1.099	2.56	0.010
Stage of disease Stage 0 versus I Stage 0 versus II Stage 0 versus III and IV	3.00 3.76 5.84	1.773 2.359 4.257	0.943, 9.554 1.102, 12.858 1.399, 24.372	1.86 2.12 2.42	0.063 0.034 0.015
SLNB	0.40	0.137	0.206, 0.784	−2.67	0.007
Number of nodes removed	1.08	0.021	1.043, 1.126	4.10	<.0001
Any chemotherapy	1.20	0.368	0.654, 2.184	0.58	0.561
Any radiation therapy	1.32	0.390	0.738, 2.356	0.93	0.351
Self-reported race/ethnicity White versus Black White versus Asian/Pacific Islander White versus Hispanic/Mixed	4.35 2.90 2.35	4.880 2.693 1.290	0.483, 39.201 0.472, 17.875 0.798, 6.890	1.31 1.15 1.55	0.190 0.250 0.121
Genomic race/ethnicity AIMS – Principal component 1 AIMS – Principal component 2 AIMS – Principal component 3	0.75 0.86 1.10	0.121 0.104 0.122	0.549, 1.030 0.678, 1.090 0.886, 1.367	−1.78 −1.25 0.87	0.076 0.211 0.386
Overall model fit: χ^2^ = 77.83, p<0.0001, R^2^ = 0.1735					

Abbreviations: AIMS  =  ancestry informative markers; BMI  =  body mass index; CI  = confidence interval; SLNB  =  sentinel lymph node biopsy.

In the second regression analysis that added genomic estimates of and self-reported race/ethnicity in addition to the characteristics listed above, the overall model explained 17.4% of the variance in LE group membership (p<0.0001). While BMI, stage of disease, SLNB status, and number of lymph nodes removed remained significant, when genomic and self-reported race/ethnicity were included in the model, the receipt of CTX and RT were no longer significant predictors of LE group membership.

### Candidate gene analyses for the development of LE

As summarized in Table 1, no associations with the occurrence of LE were found in the SNPs evaluated for HGF, LYVE1, MET, PROX1, RORC, VEGFB, VEGFD, and VEGFR3. However, the genotype frequency was significantly different between those who did and did not develop LE for thirteen SNPs and seven haplotypes spanning nine genes (i.e., ANGPT2, FOXC2, LCP2, NRP2, SOX 17, SYK, VCAM1, VEGFC, and VEGFR2). For the SNP in ANGPT2 (rs6990020), a dominant model fit the data best (p = .040). One haplotype (HapA03, p = .045) was identified in FOXC2. For the three SNPs (rs572192, rs315721, rs6866733) identified in LCP2, a dominant model fit the data best (p = .047, p = .005, p = .026, respectively). Three SNPs (rs849530, rs849563, rs16837641) and one haplotype (HapF01, p = .011) were identified in NRP2. For all three SNPs, a recessive model fit the data best (p = .042, p = .012, p = .009, respectively). For the SNP in SOX17 (rs12541742), a dominant model fit the data best (p = .008). One SNP (rs158689) and 1 haplotype (HapD01, p = .008) were identified in SYK. For rs158689, a recessive model fit the data best (p = .039). One SNP (rs3176861) and one haplotype (HapB01, p = .016) were identified in VCAM1. For rs3176861, a dominant model fit the data best (p = .036). One SNP (rs7664413) and two haplotypes (HapA05, p = .046; HapB03, p = .027) were identified in VEGFC. For rs7664413, an additive model fit the data best (p = .041). Two SNPs (rs10020464, rs11133360) and one haplotype (HapC04, p = .021) were identified for VEGFR2. For rs10020464, a dominant model fit the data best (p = .025) and for rs11133360 a recessive model fit the data best (p = .032).

### Regression analyses of FOXC2, LCP2, NRP2, SYK, VCAM1, and VECFC genotypes and haplotypes and the development of LE

In order to better estimate the magnitude (i.e., odds ratio, OR) and precision (95% confidence interval, CI) of genotype on the development of LE, multivariate logistic regression models were fit. As shown in Table 4, in addition to genotype, the phenotypic variables included in the regression models were ethnicity (i.e., White, Black, Asian, Hispanic/Mixed ethnic background/Other), BMI, stage of disease, having a SLNB, and number of lymph nodes removed. Receipt of CTX and RT, while not significant after the inclusion of genomic estimates of and self-reported race/ethnicity, were retained in all of the regression models for face validity.

**Table 4:** Multiple logistic regression analyses for FOXC2. LCP2, NRP2, SYK, VCAM1, and VEGF-C genotypes and halotypes to predict the development of lymphedema.

Predictor	Odds Ratio		Standard Error			95% CI				Z			p-value
FOXC2 haplotype	0.63		0.125			0.430, 0.931				−2.32			0.020
BMI	1.06		0.022			1.016, 1.104				2.72			0.006
Stage of disease Stage 0 versus I Stage 0 versus II Stage 0 versus III and IV	3.43 4.10 6.71		2.053 2.619 4.975			1.060, 11.085 1.175, 14.333 1.571, 28.687				2.06 2.21 2.57			0.040 0.027 0.010
SLNB	0.40		0.138			0.203, 0.784				−2.67			0.008
Number of nodes removed	1.08		0.022			1.042, 1.127				4.01			<.0001
Any chemotherapy	1.22		0.380			0.664, 2.246				0.64			0.521
Any radiation therapy	1.35		0.406			0.752, 2.436				1.01			0.313
Overall model fit: χ^2^ = 83.44, p<0.0001, R^2^ = 0.1860													
LCP2 genotype	0.50		0.132				0.298, 0.838				−2.63	0.009	
BMI	1.05		0.022				1.012, 1.097				2.54	0.011	
Stage of disease Stage 0 versus I Stage 0 versus II Stage 0 versus III and IV	3.01 3.56 5.94		1.788 2.238 4.365				0.937, 9.643 1.040, 12.203 1.407, 25.079				1.85 2.02 2.42	0.064 0.043 0.015	
SLNB	0.40		0.137				0.204, 0.784				−2.67	0.008	
Number of nodes removed	1.08		0.022				1.040, 1.125				3.93	<.0001	
Any chemotherapy	1.25		0.389				0.679, 2.298				0.71	0.475	
Any radiation therapy	1.33		0.398				0.744, 2.392				0.97	0.334	
Overall model fit: χ^2^ = 84.86, p<0.0001, R^2^ = 0.1891													
NRP2 genotype	0.38		0.143			0.185, 0.799				−2.56		0.010	
BMI	1.06		0.023			1.016, 1.105				2.68		0.007	
Stage of disease Stage 0 versus I Stage 0 versus II Stage 0 versus III and IV	2.79 3.41 6.01		1.647 2.150 4.430			0.874, 8.875 0.990, 11.733 1.420, 25.477				1.73 1.94 2.44		0.083 0.052 0.015	
SLNB	0.39		0.136			0.203, 0.775				−2.71		0.007	
Number of nodes removed	1.08		0.021			1.040, 1.122				4.00		<0.0001	
Any chemotherapy	1.18		0.369			0.635, 2.174				0.51		0.607	
Any radiation therapy	1.20		0.363			0.667, 2.171				0.61		0.539	
Overall model fit: χ^2^ = 85.11, p<0.0001, R^2^ = 0.1897													
NRP2 haplotype		0.54			0.114	0.358, 0.817		−2.92				0.003	
BMI		1.05			0.022	1.011, 1.099		2.48				0.013	
Stage of disease Stage 0 versus I Stage 0 versus II Stage 0 versus III and IV		3.03 3.81 5.49			1.805 2.433 4.081	0.941, 9.738 1.088, 13.321 1.281, 23.561		1.86 2.09 2.29				0.063 0.036 0.022	
SLNB		0.40			0.137	0.201, 0.780		−2.68				0.007	
Number of nodes removed		1.09			0.021	1.046, 1.132		4.21				<0.0001	
Any chemotherapy		1.11			0.348	0.602, 2.054		0.34				0.734	
Any radiation therapy		1.30			0.391	0.723, 2.346		0.88				0.379	
Overall model fit: χ^2^ = 86.91, p<0.0001, R^2^ = 0.1937													
SYK genotype	3.43		1.131			1.797, 6.546		3.74				<0.0001	
BMI	1.06		0.023			1.017, 1.106		2.76				0.006	
Stage of disease Stage 0 versus I Stage 0 versus II Stage 0 versus III and IV	3.05 3.10 5.50		1.821 1.968 4.078			0.946, 9.832 0.891, 10.760 1.286, 23.523		1.87 1.78 2.30				0.062 0.075 0.022	
SLNB	0.40		0.141			0.201, 0.797		−2.61				0.009	
Number of nodes removed	1.10		0.022			1.054, 1.142		4.53				<0.0001	
Any chemotherapy	1.44		0.459			0.773, 2.692		1.15				0.249	
Any radiation therapy	1.45		0.437			0.803, 2.616		1.23				0.218	
Overall model fit: χ^2^ = 91.90, p<0.0001, R^2^ = 0.2049													
VCAM1 genotype	0.55			0.158		0.309, 0.963			−2.09			0.037	
BMI	1.06			0.022		1.015, 1.102			2.68			0.007	
Stage of disease Stage 0 versus I Stage 0 versus II Stage 0 versus III and IV	2.85 3.78 5.92			1.710 2.413 4.385		0.879, 9.239 1.080, 13.214 1.388, 25.275			1.75 2.08 2.40			0.081 0.038 0.016	
SLNB	0.41			0.141		0.206, 0.801			−2.60			0.009	
Number of nodes removed	1.08			0.022		1.043, 1.128			4.07			<0.0001	
Any chemotherapy	1.18			0.365		0.643, 2.161			0.53			0.596	
Any radiation therapy	1.30			0.387		0.724, 2.327			0.88			0.381	
Overall model fit: χ^2^ = 82.35, p<0.0001, R^2^ = 0.1836													
VEGFC haplotype	0.64		0.145			0.409, 0.997		−1.97				0.049	
BMI	1.05		0.022			1.011, 1.097		2.46				0.014	
Stage of disease Stage 0 versus I Stage 0 versus II Stage 0 versus III and IV	2.94 3.74 5.42		1.740 2.351 3.962			0.920, 9.380 1.091, 12.820 1.291, 22.716		1.82 2.10 2.31				0.069 0.036 0.021	
SLNB	0.42		0.145			0.215, 0.826		−2.51				0.012	
Number of nodes removed	1.09		0.021			1.044, 1.128		4.16				<0.0001	
Any chemotherapy	1.24		0.387			0.675, 2.287		0.70				0.487	
Any radiation therapy	1.31		0.391			0.729, 2.354		0.90				0.366	
Overall model fit: χ^2^ = 81.85, p<0.0001, R^2^ = 0.1825													

For each model, the first three principal components identified from the analysis of ancestry informative markers as well as self-report race/ethnicity (i.e., White, Black, Asian/Pacific Islander, Hispanic/Mixed ethnic background/Other) were retained in all models to adjust for potential confounding due to race or ethnicity (data not shown). Predictors evaluated in each model included genotype (FOXC2 haplotype A03 composed of the rs34221221 “C” allele and the rs1035550 “C” allele; LCP2 rs315721: AA versus AG+GG; NRP2 rs849530: TT+TG versus GG; NPR2 haplotype F01 composed of the rs849530 “G” allele, the rs950219 “G” allele, and the rs3771052 “G” allele; SYK rs158689: AA+AT versus TT; VCAM1 rs3176861: CC versus CT + TT; VEGFC haplotype B03 composed of the rs3775202 “G” allele and the rs3775195 “C” allele), BMI (kilograms/meter squared), stage of disease, SLNB, number of lymph nodes removed, receipt of chemotherapy prior to or following surgery, and receipt of radiation therapy following surgery.

Abbreviations: BMI  =  body mass index; CI  = confidence interval; FOXC2  =  Forkhead box protein C2; LCP2  =  Lymphocyte cytosolic protein 2; NRP  =  neuropilin-2; SLNB  =  sentinel lymph node biopsy; SYK  =  spleen tyrosine kinase; VCAM1  =  vascular cell adhesion molecule 1; VEGFC  =  vascular endothelial growth factor-C.

**Figure 1:**
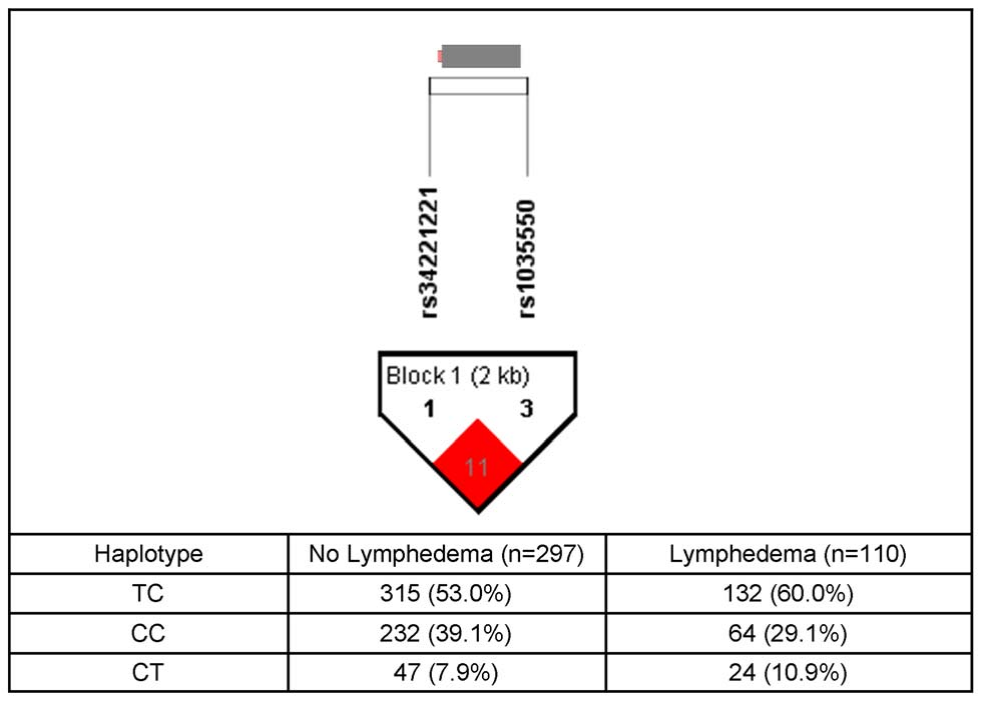
FOXC2 Gene Structure and Linkage Disequilibrium. An ideogram of forkhead box C2 (FOXC2) is presented above the white bar that represents the physical distance along human chromosome 16 (chr16: 85,158,358–85,160,040; genome assembly 36.3, NM_005251.2). Exons are represented as thick bars. Reference sequence identifiers (rsID) for each single nucleotide polymorphism (SNP) are plotted both in terms of their physical distance (i.e., the white bar at the top of the figure) and equidistantly to render the pairwise linkage disequilibrium (LD) estimates that were calculated and visualized with Haploview 4.2. The gene structure for FOXC2 was rendered with FancyGene 1.4. The correlation statistic (r^2^ and D') is provided in the heatmap. LD-based haplotype block definition was based on the D' confidence interval method. The haploblock is indicated in a bolded triangle and its component SNPs are rendered in bold font. Pairwise D' value (range: 0–1, inclusive) was rendered in color, with darker red diamond representing D' value approaching 1.0. When the r^2^ value (range of 0–100, inclusive) is not equal to 0 or 100, it is provided in a given diamond. The 2-SNP haplotype associated with LE is composed of one rare and one common allele of two SNPs located in the immediate early promoter (rs34221221; rare “C” allele) and immediately downstream of the FOXC2 coding region (rs1035550; common “C” allele).

**Figure 2:**
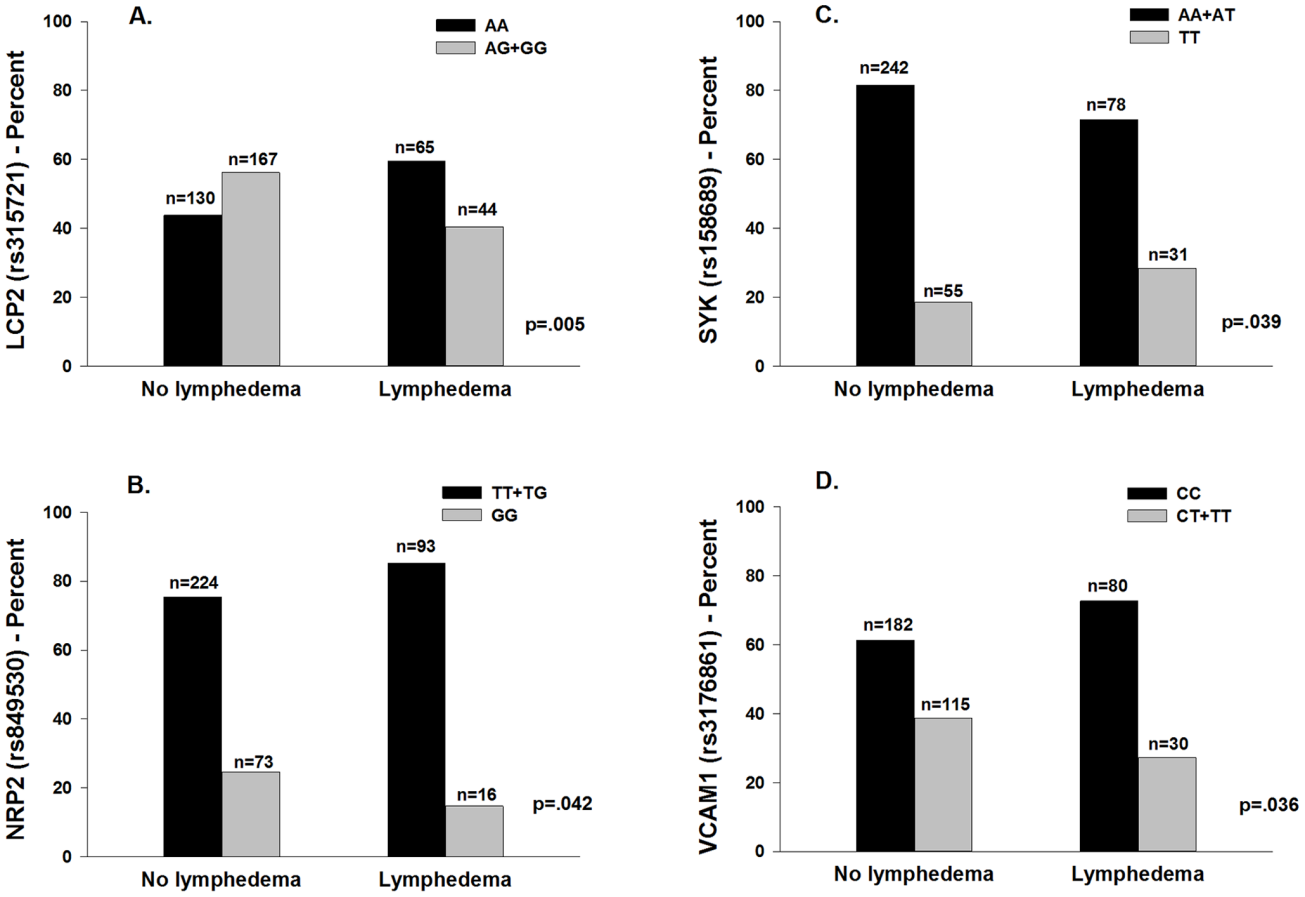
Differences between the lymphedema and no lymphedema groups. A – Differences between the lymphedema and no lymphedema groups in the percentages of patients who were homozygous for the common allele (AA) or heterozygous or homozygous for the rare allele (AG+GG) for rs315721 in lymphocyte cytosolic protein 2 (LCP2). B – Differences between the lymphedema and no lymphedema groups in the percentages of patients who were homozygous or heterozygous for the common allele (TT+TG) or homozygous for the rare allele (GG) for rs849530 in neuropilin-2 (NRP2). C – Differences between the lymphedema and no lymphedema groups in the percentages of patients who were homozygous or heterozygous for the common allele (AA+AT) or homozygous for the rare allele (TT) for rs158689 in protein tyrosine kinase (SYK). D – Differences between the lymphedema and no lymphedema groups in the percentages of patients who were homozygous for the common allele (CC) or heterozygous or homozygous for the rare allele (CT+TT) for rs3176861 in vascular cell adhesion molecule 1 (VCAM1).

**Figure 3:**
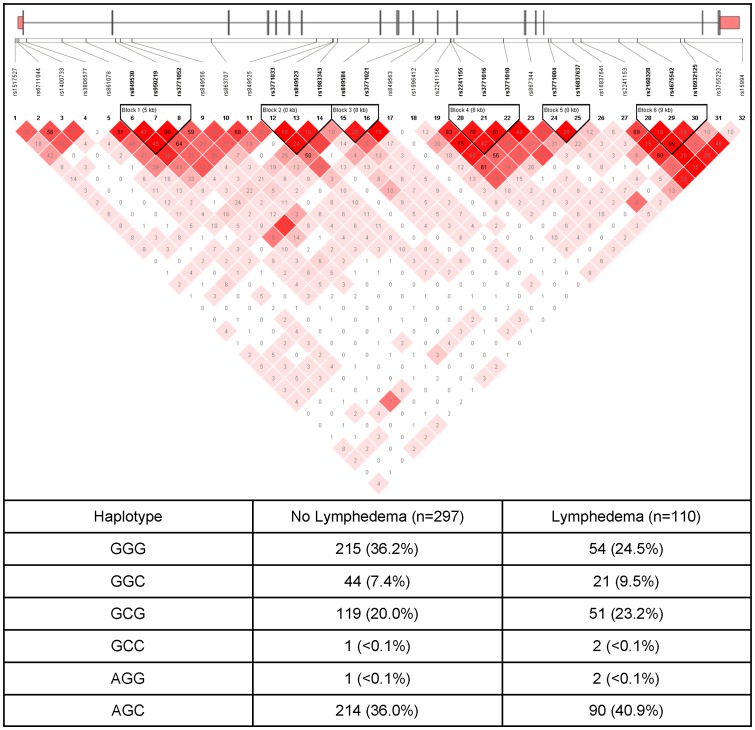
NRP2 Gene Structure and Linkage Disequilibrium. An ideogram of neuropilin 2 (NRP2) is presented above the white bar that represents the physical distance along human chromosome 2 (chr2: 206,255,469–206,371,102; genome assembly 36.3, NM_201266.1). Exons are represented as thick bars. Gray lines connecting the exons represent introns. Reference sequence identifiers (rsID) for each single nucleotide polymorphism (SNP) are plotted both in terms of their physical distance (i.e., the white bar at the top of the figure) and equidistantly to render the pairwise linkage disequilibrium (LD) estimates that were calculated and visualized with Haploview 4.2. The gene structure for NRP2 was rendered with FancyGene 1.4. The correlation statistics (r^2^ and D') are provided in the heatmap. LD-based haplotype block definition was based on the D' confidence interval method. The haploblock is indicated in a bolded triangle and its component SNPs are rendered in bold font. Pairwise D' values (range: 0–1, inclusive) were rendered in color, with darker red diamonds representing D' values approaching 1.0. When the r^2^ values (range of 0–100, inclusive) are not equal to 0 or 100, they are provided in a given diamond. The 3-SNP haplotype associated with LE consists of one rare and two common alleles of three SNPs (rs849530 “G” rare allele, rs950219 “G” common allele, rs3771052 “G” common allele) located in intron 1 of the gene.

**Figure 4:**
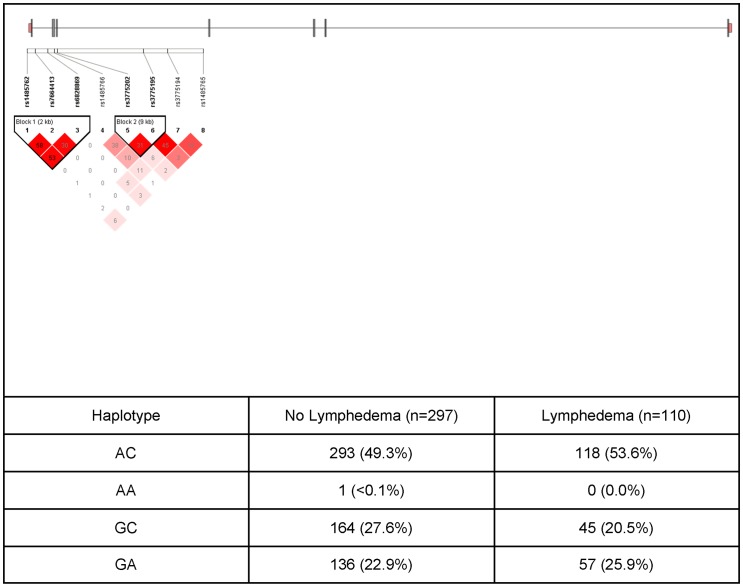
VEGFC Gene Structure and Linkage Disequilibrium. An ideogram of vascular endothelial growth factor C (VEGFC) is presented above the white bar that represents the physical distance along human chromosome 4 (chr4: 177,841,685–177,950,889; genome assembly 36.3, NM_005429.2). Exons are represented as thick bars. Gray lines connecting the exons represent introns. Reference sequence identifiers (rsID) for each single nucleotide polymorphism (SNP) are plotted both in terms of their physical distance (i.e., the white bar at the top of the figure) and equidistantly to render the pairwise linkage disequilibrium (LD) estimates that were calculated and visualized with Haploview 4.2. The gene structure for VEGFC was rendered with FancyGene 1.4. The correlation statistics (r^2^ and D') are provided in the heatmap. LD-based haplotype block definition was based on the D' confidence interval method. The haploblock is indicated in a bolded triangle and its component SNPs are rendered in bold font. Pairwise D' values (range: 0–1, inclusive) were rendered in color, with darker red diamonds representing D' values approaching 1.0. When the r^2^ values (range of 0–100, inclusive) are not equal to 0 or 100, they are provided in a given diamond. The 2-SNP haplotype associated with LE consists of one rare and one common allele of two SNPs (rs3775202 “G” rare allele, rs3775195 “C” common allele) located in intron 4 of the gene. Of note, the strong linkage disequilibrium estimates observed in public databases (i.e., HapMap) resulted in the selection of 8 SNPs that tagged the entire coding region of the VEGFC gene.

The only genetic associations that remained significant in the multivariate logistic regression analyses were for FOXC2 haplotype A03, LCP2 rs315721, NRP2 rs849530, NRP2 haplotype F01, SYK rs158689, VCAM1 rs3176861, and VEGFC haplotype B03 (see Table 4 and Figures 1, 2, 3, and 4). In the regression analysis for the FOXC2 haplotype A3, which was composed of two SNPs (i.e., rs34221221, rs1035550), the overall model explained 18.6% of the variance in the odds of developing LE. Each additional dose of the FOXC2 haplotype A03 was associated with 37.0% decrease in the odds of developing LE. Figure 1 displays the FOXC2 LD-based heatmap and haplotype analysis.

In the regression analysis for LCP2 rs315721, the overall model explained 18.9% of the variance in the odds of developing LE. Carrying one or two doses of the rare allele (i.e., AA versus AG + GG) was associated with a 50.0% decrease in the odds of developing LE (Figure 2A).

In the regression analysis for NRP2 rs849530, the overall model explained 19.0% of the variance in the odds of developing LE. Carrying two doses of the rare allele (i.e., TT+TG versus GG) was associated with 62.0% decrease in the odds of developing LE (Figure 2B). In the regression analysis for the NRP2 haplotype F01, which was composed of three SNPs (i.e., rs849530, rs950219, rs3771052), the overall model explained 19.4% of the variance in the odds of developing LE. Each additional dose of the NRP2 haplotype F01 was associated with 46.0% decrease in the odds of developing LE. Figure 3 displays the NRP2 LD-based heatmap and haplotype analysis.

In the regression analysis for SYK rs158689, the overall model explained 20.1% of the variance in the odds of developing LE. Carrying two doses of the rare allele (i.e., AA + AT versus TT) was associated with 3.43-fold increase in the odds of developing LE (Figure 2C).

In the regression analysis for VCAM1 rs3176861, the overall model explained 18.4% of the variance in the odds of developing LE. Carrying one or two doses of the rare allele (i.e., CC versus CT + TT) was associated with a 45.0% decrease in the odds of developing LE (Figure 2D).

In the regression analysis for the VEGFC haplotype B03, which was composed of two SNPs (i.e., rs3775202, rs3775195), the overall model explained 18.3% of the variance in the odds of developing LE. Each additional dose of the VEGFC haplotype B03 was associated with 36.0% decrease in the odds of developing LE. Figure 4 displays the VEGFC LD-based heatmap and haplotype analysis.

## Discussion

This study is the first to evaluate phenotypic and genotypic predictors of LE in a large cohort of women who had LE diagnosed using BIS rather than self-report. Based on an extensive evaluation of demographic, disease, and treatment characteristics, the factors associated with an increased risk of LE in the bivariate analyses were higher BMI, poorer functional status, having lung disease, a higher stage of disease, increased number of lymph nodes removed, increased number of positive lymph nodes, not having a SLND, having had an ALND, and receipt of CTX or RT. All of these risk factors are consistent with previous reports.

In the initial multivariate analyses, poorer functional status, having lung disease, number of positive nodes, and having had an ALND were not retained in the final model (Table 3). However, when genomic estimates of and self-reported race/ethnicity were added to the multivariate logistic regression analysis, receipt of CTX and RT were no longer significant predictors of LE group membership. This final phenotypic model explained only 17.4% of the variance in LE group membership. These findings suggest that complex interactions may exist between phenotypic characteristics and the development of LE. Future studies of LE risk need to evaluate a comprehensive list of phenotypic characteristics as well as interactions among these characteristics.

In the past few years, the complex array of molecular events that regulate the development and maintenance of lymphatic system, as well as contribute to its malfunction have begun to be elucidated in animals and humans [10], [11], [13]. In this study, candidate genes were selected that were identified in previous animal and human studies to play a role in lymphatic morphogenesis (Table 1). While not all of the candidate genes were associated with the development of LE, the significant associations that were identified provide new information on genomic risk factors and potential therapeutic targets. Of note, each of the SNPs explained between 1.0% (VCAM1 rs3176861) to 3.1% (SYK rs158689) of the variance in the development of LE.

FOXC2 is a transcription factor that appears to be important for the normal development and maintenance of both venous and lymphatic vessels [45]. In adults, FOXC2 is highly expressed in developing lymphatic vessels as well as in lymphatic valves [46], [47]. While early lymphatic development will proceed normally in the absence of FOXC2, the collecting lymphatic vessels that are formed lack valves and the lymphatic capillaries acquire an ectopic coverage of basement membrane components and smooth muscle cells [46]. FOXC2 is the transcription factor associated with lymphedema-distichiasis (LD, OMIM #153400) a monogenic disorder that is characterized by late onset LE, a double row of eyelashes, and varicose veins [48]–[50]. Therefore, common functional polymorphisms that result in modest alterations in function or expression of the FOXC2 transcription factor may be associated with the development of secondary LE following breast cancer treatment. The FOXC2 A03 haplotype consists of one rare and one common allele in two SNPs located in the immediate early promoter (rs34221221; rare “C” allele) and immediately downstream from the FOXC2 coding region (rs1035550, common “C” allele). SNP rs34221221 occurs in the immediate early promoter of the FOXC2 gene (−514) which is a highly conserved region of the gene. While functional studies need to be done, it is reasonable to suggest that this polymorphism alters transcription factor binding and subsequent gene expression. SNP rs103550 lies downstream from the FOXC2 coding region of the gene and is not likely to have a functional role. Functional studies of this two-SNP haplotype are warranted if the association with LE is replicated in an independent sample.

LCP2 functions in lymphatic vessel development by modulation of the hematopoetic signaling pathway that mediates the separation of the two major vascular networks (i.e., blood, lymphatic) [51]. SYK acts on LCP2 as part of a central signaling pathway that regulates separation of these two vascular networks. Variation in either locus could result in their altered interaction with upstream (i.e., SYK) or downstream (i.e., LCP2) members of the signaling cascade. Mice with mutations in SYK develop arterio-venous shunts and abnormal lymphatic-venous connections [51], [52]. In addition, genetic ablation of SYK causes the accumulation of leukocytes that is associated with lymphatic proliferation and lymphatic vessel dilation which results in the formation of shunts between the blood and lymphatic vessels (F. Kiefer, personal communication cited in Tammela and Alitalo [12]). While LPC2 rs35721 is located in the intronic region of the gene and has no known function, it could be in LD with a functional variation. Although SYK rs158689 is located in an intron, it is predicted to disrupt a putative CCCTC-binding factor (CTCF) termed insulator elements. These insulator elements play a vital role in the regulation of gene expression by limiting the boundary of heterochromatin and by restricting transcriptional factor access [53]. Functional studies are needed to determine if the rare “T” allele of rs158689 alters the function of the putative insulator element and SYK gene expression.

Lymphatic vessels participate in inflammatory responses by promoting lymphocyte transport to draining lymph nodes [54]. VCAM1 plays a role in the migration of lymphocytes into lymphoid organs. Compared to healthy controls, cytokines levels are increased in lymphatic fluid from patients with obstructive LE. These elevated levels of cytokines may be due to their local production by infiltrating immune cells [55]. The elevated levels of these cytokines would be expected to contribute to the chronic inflammation that is typically observed in tissues with poor lymphatic drainage. Allelic variations in VCAM1 could influence the rate of lymphocyte homing to lymph nodes. Like other SNPs in this study, rs3176861, located in the intron of the VCAM gene, has no known function. However, it could be in LD with a functional variation.

NRP1 and NRP2 are transmembrane proteins involved in a number of physiologic processes [56]–[59]. NRP2 can bind to members of the VEGF family of growth factors and influence lymphagiogenesis. NRP2 is expressed in a subset of lymphatic vessels and acts as a co-receptor for VEGFC [60]. VEGFC binds to and activates VEGFR3 and VEGFR2 receptors on the lymphatic epithelium [61]–[65]. In one study [66], *Nrp2* knockout mice did not exhibit defects in blood vessels. However, lymphatic development was abnormal, including abnormal patterning and marked reduction in small lymphatic vessels and capillaries. These findings suggest that NRP2 has a role in VEGFC mediated VEGFR3 signaling and lymphangiogenesis. The NRP2 haplotype consists of one rare and two common alleles in three SNPs (rs849530 “G” rare allele, rs950219 “G” common allele, rs3771052 “G” common allele) located in intron 1 of the gene. These SNPs lie in a conserved region of the NRP2 gene. However, this haplotype has no known function which suggest that this haplotype is a surrogate for an unmeasured SNP(s) in LD with the haplotype. The VEGFC haplotype consists of one rare and one common allele in two SNPs (rs3775202 “G” rare allele, rs3775195 “C” common allele) located in intron 4 of the gene. These SNPs lie in a conserved region of the VEGFC gene. However, this haplotype has no known or predicted function which suggests that it is a surrogate for an unmeasured SNP(s) in LD with this haplotype.

It is interesting to note that no associations were found in SNPs for HGF, LYVE1, MET, PROX1, RORC, VEGFB, VEGFC, and VEGFR3. One possible reason for this lack of association is that these genes are involved primarily during embryogenesis in the development of the lymphatic system and are not involved in the mechanisms that underlie the development of secondary LE [10], [11], [13]. However, recent evidence suggests that the VEGF pathway is involved in lymphangiogenesis associated with inflammation [12], [67]. An alternative explanation for the lack of association is that the occurrence of the minor allele was too infrequent in this sample to identify significant differences between patients with and without LE. Evidence to support this hypothesis is found in Table 1, where p-values for some of these genes approached statistical significance.

Several study limitations need to be acknowledged. While the sample size was relatively large, larger samples may identify additional candidate gene associations. In terms of the genetic analyses, additional studies are needed to confirm the associations found in this study. Future studies can evaluate additional candidate genes or perform genome wide association studies to uncover novel molecular pathways. Once candidate genes are confirmed, then DNA sequencing may need to be performed to find the causal variant(s). Despite these limitations, findings from this study suggest a role for a number of lymphatic and angiogenic candidate genes in the development of secondary LE following breast cancer treatment.
